# Management of liver metastases from non-functional gastroenteropancreatic neuroendocrine tumors: a systematic review

**DOI:** 10.3389/fendo.2025.1601185

**Published:** 2025-07-24

**Authors:** Jun-shuai Xue, Yi Yang, Zhen Huang, Hong Zhao, Xiao Chen, Jian-qiang Cai

**Affiliations:** Department of Hepatobiliary Surgery, National Cancer Center/Cancer Hospital, Chinese Academy of Medical Sciences and Peking Union Medical College, Beijing, China

**Keywords:** gastroenteropancreatic neuroendocrine tumors, liver metastasis, treatment, multidiscipline, review

## Abstract

The liver is the most common metastatic organ of neuroendocrine tumors (NETs). NET liver metastases (NETLMs) are categorized into simple liver metastasis (type I), complex liver metastasis (type II) and diffuse liver metastasis (type III), of which diffuse liver metastasis accounts for the highest percentage, up to 60-70%. Radical resection is recommended for all patients with type I and partial type II liver metastases without extrahepatic metastases in G1 and G2 grades, with a 5-year survival rate of 65%-70%. But for patients with G3 or type III liver metastases, treatment is controversial. Ablation and TAE/TACE are commonly used localized treatments. Somatostatin analogue (octreotide and lanreotide) are efficacious in the treatment of better-differentiated NETs and can prolong the progression-free survival (PFS) of patients. Targeted drugs such as sunitinib, everolimus, sofantinib and cabozantinib are used to control tumor growth and improve symptoms. In addition, peptide receptor radionuclide therapy (PRRT), has been approved by the FDA for the treatment of progressive somatostatin receptor-positive gastroenteropancreatic NETs and has shown potential for prolonging PFS and improving survival. Multidisciplinary treatment is crucial for patients with NETLMs with high tumor load, and neoadjuvant therapy combined with surgery may lead to a better prognosis. However, the choice of treatment, indications for combination therapy, and disease prognosis still require further research and exploration. This review summarizes and evaluates the current treatment strategies and development trend of NETLM treatment through a literature review and provides new ideas as well as insights.

## 1 Introduction

Neuroendocrine tumors (NETs) are a heterogeneous group of tumors that originate from neuroendocrine cells and can occur in different organs, frequently located in the gastroenteropancreas (GEP), accounting for about 55%-70% (1). According to data from national databases and registries, the incidence of GEP-NET in the United States (US) was approximately 3.56/100,000; the incidence of NET in the United Kingdom in 2018 was approximately 9/100,000; and the incidence of GEP-NET in Japan in 2016 was 3.53/100,000. The incidence of pancreatic NET (pNET) and rectal NET has increased more significantly (2, 3). According to the US surveillance, epidemiology and database, the incidence and prevalence of NENs has increased significantly, with their incidence rising sixfold over the past 40 years (4). The WHO (2022) classifies them into well-differentiated neuroendocrine tumors (G1, G2 and G3), poorly differentiated neuroendocrine carcinomas (including large and small cell types), and mixed neuroendocrine-nonneuroendocrine tumors, based on the nuclear fission image and the Ki-67 index (5).

**Figure 1:**
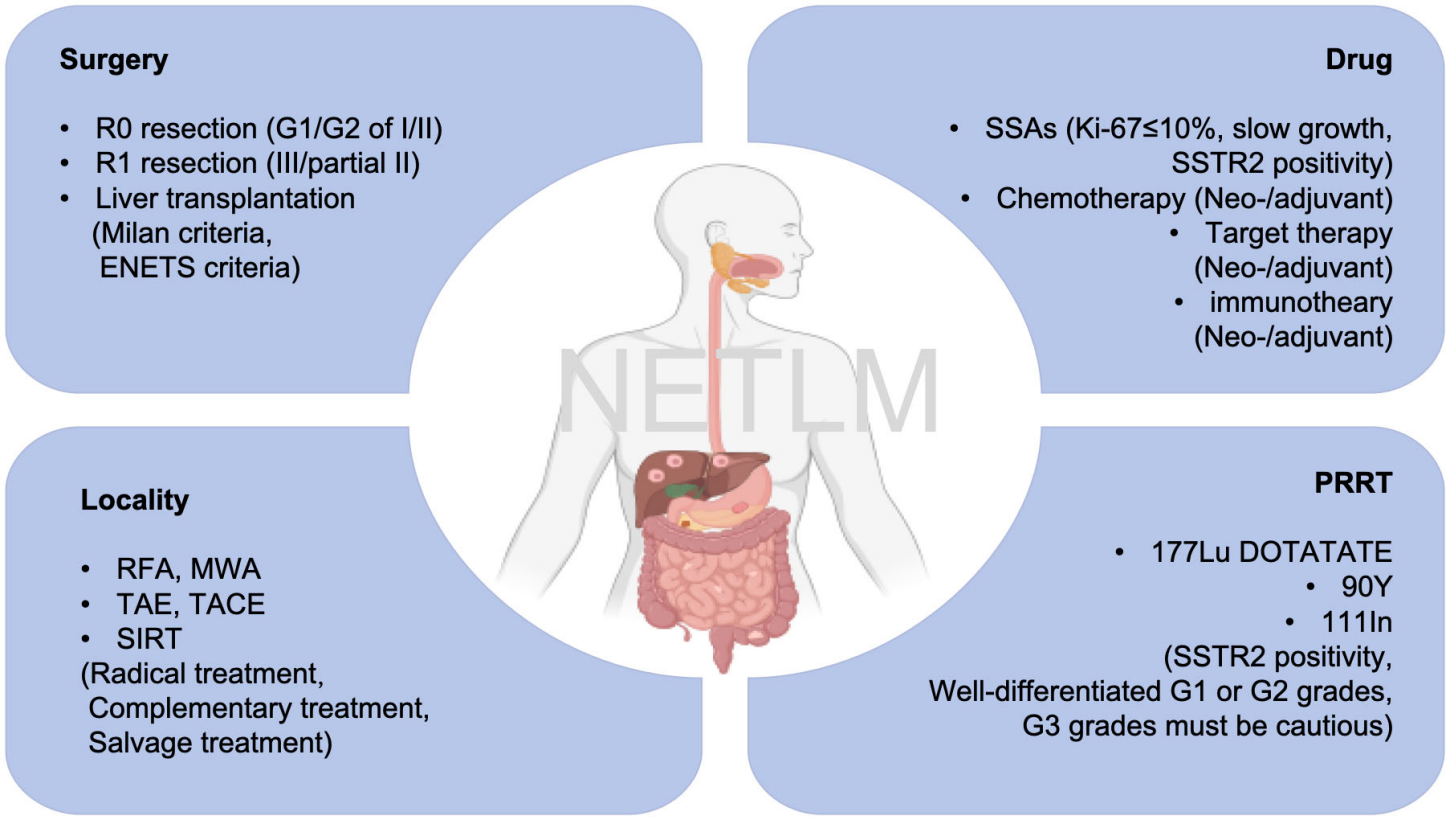
The treatment and indications for NETLM patients.

Liver is the most common metastatic organ. The European Neuroendocrine Tumor Society (ENETS) guidelines classify NET liver metastases (NETLMs) into 3 types. First, simple LMs, which account for 20% to 25% of cases, are confined to one liver lobe or two adjacent resectable liver segments. The second is complex LMs, accounting for 10% to 15% of cases, i.e., one large metastasis in one hepatic lobe and multiple small metastases in the contralateral hepatic lobe, with the possibility of surgical resection. Third, diffuse LMs, which account for 60% to 70% of cases, have diffuse multiple metastases in the liver that cannot be surgically resected (6). Approximately 28-77% of patients develop LMs during their lifetime, and these are also an indicator of their poor prognosis (7). Without treatment, the 5-year survival rate is about 20%-40% (8). However, according to guidelines of ENETS and the North American Neuroendocrine Society (NANETS), surgery remains the preferred treatment option for resectable patients. The 5-year survival rate of NETLM patients after surgical resection has been reported to be approximately 60-90%, but short-term recurrence of the disease remains a troubling problem (9). For NETLM with multifocal hepatic lobe involvement, numerous patients may not meet surgical criteria. Systemic therapies, including chemotherapy (etoposide, capecitabine), somatostatin analogue (e.g., octreotide, lanreotide), targeted therapies (sunitinib, everolimus, sofentinib), peptide receptor-radionuclide therapy (PRRT, 177Lu-DOTATATE, 90Y-DOTATATE and 111In), and localized therapies, e.g., ablation, transcatheter arterial embolization (TAE), transcatheter arterial chemoembolization (TACE), and selective internal radiation therapy (SIRT) are available. (**Figure 1**) With the deepening of the multidisciplinary treatment (MDT) concept, the combination of these therapeutic modalities has brought light to patients, and it is possible to achieve complete R0 resection through tumor regression after comprehensive preoperative treatment. Relevant studies have shown that there is a survival benefit for patients, even if R1 resection or tumor reduction surgery (10, 11).

Liver metastasis is a common progression pattern of GEP-NETs, and its treatment strategies remain controversial. This systematic review synthesizes evidence on surgical resection, local therapies, systemic treatments, and multidisciplinary management for NF-GEP-NETLM, aiming to clarify the indications and efficacy of different therapeutic modalities.

## 2 Methods

**Figure 2:**
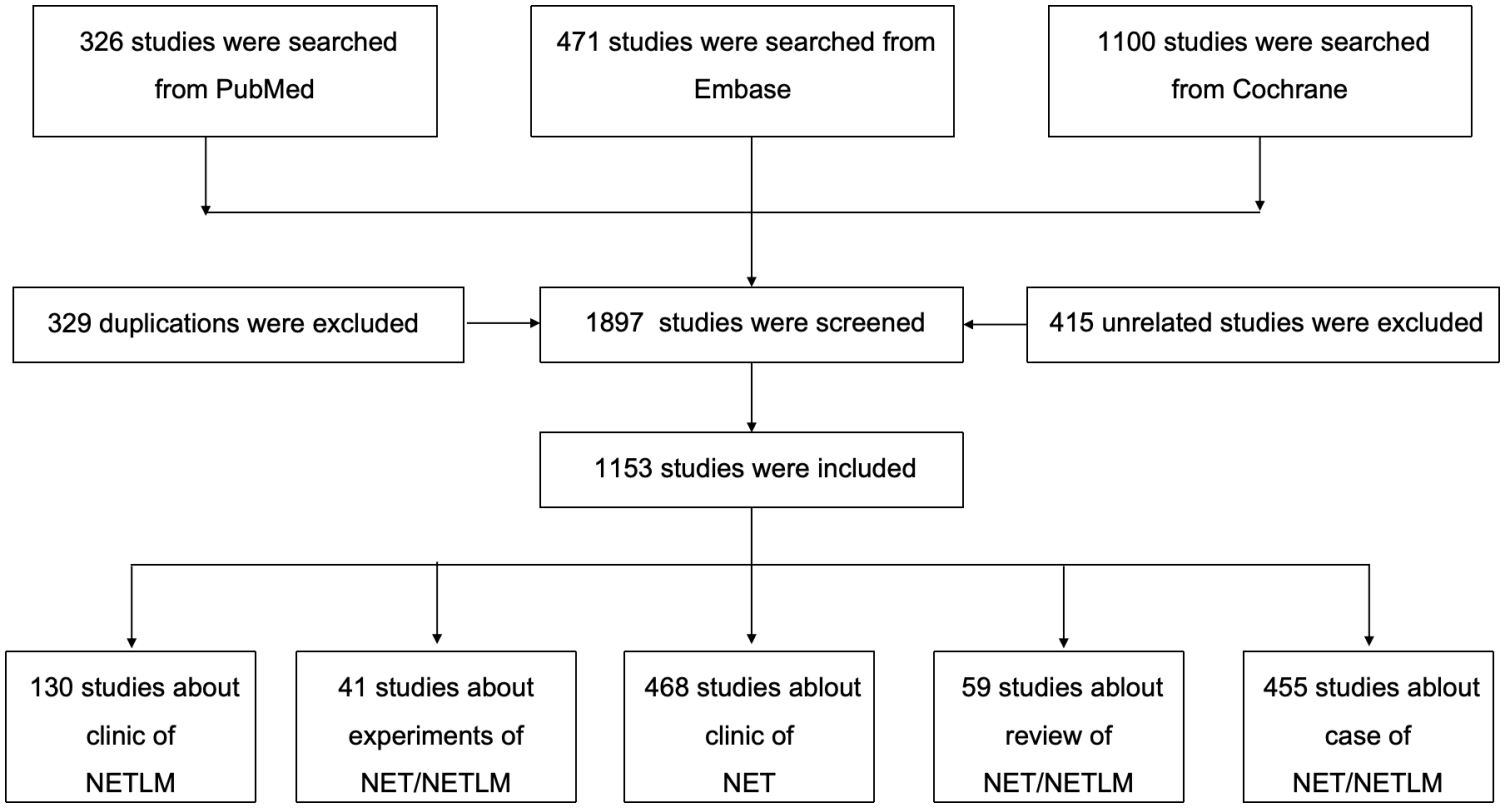
The flow chart of screening for studies.

This study was searched from PubMed, Embase, and Cochrane databases based on the search terms ((neuroendocrine tumor [Title/Abstract]) OR (neuroendocrine neoplasm [Title/Abstrac])) AND (liver metastasis [Title/Abstract)]). The language is English. The cut-off date was from the inception of the database to October 20, 2024, and the screening strategy follows the PRISMA standard and can be found in **Figure 2**; **Supplementary Table 1**.

## 3 Results

**Figure 3:**
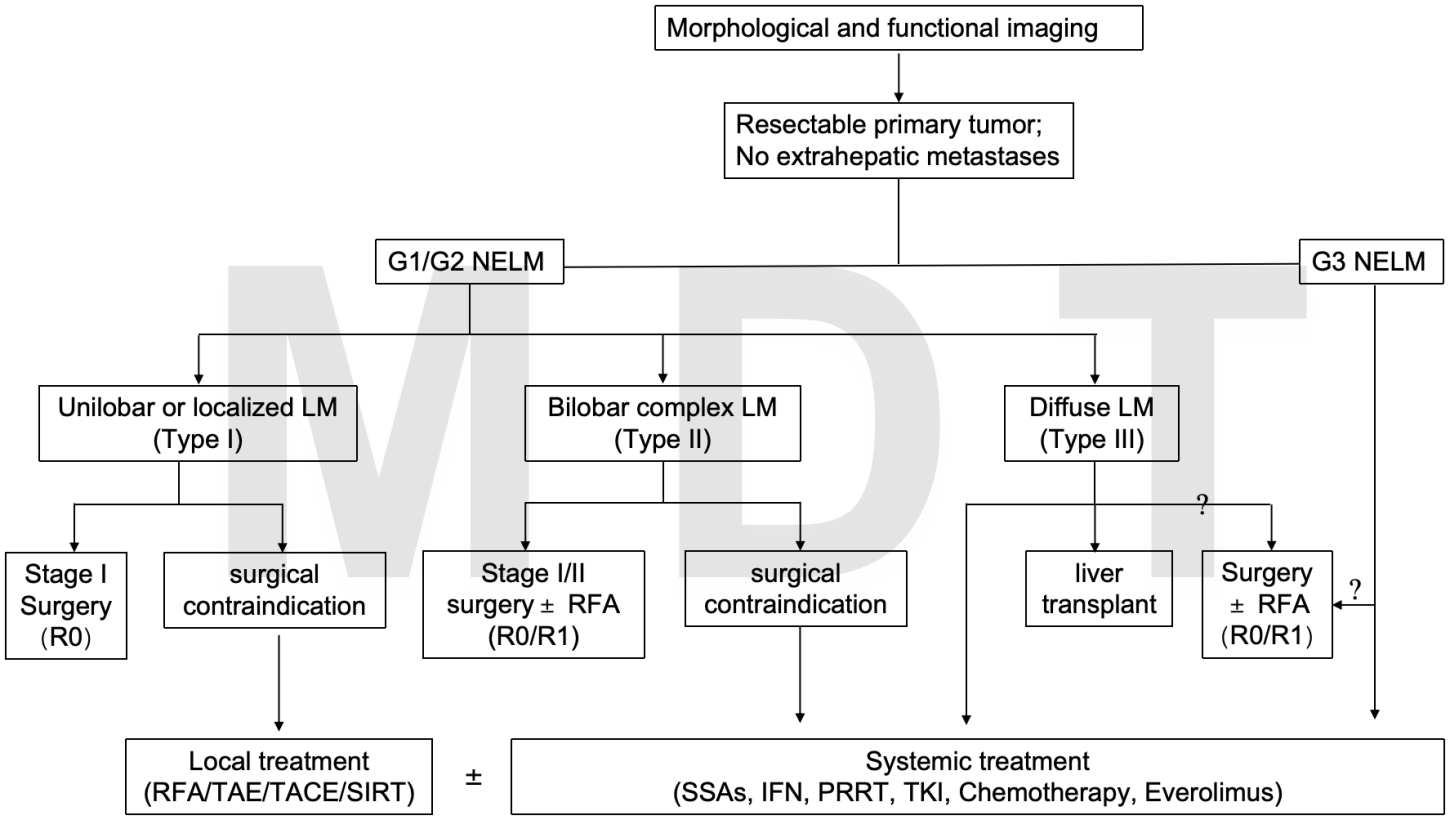
The diagram of the proposed treatments for NETLM.

The diagram of the proposed treatments for NETLM was shown in **Figure 3**.

### 3.1 Surgery

Patients with type I LMs without extrahepatic metastases in G1 and G2 grades are recommended to undergo radical resection, which can maximize the survival benefit (12). The 5-year overall survival (OS) of radical resection is 65%-70% (13). For patients with partial type II, surgical resection remains the mainstay of treatment for NETLM. Related studies have shown similar survival outcomes after R0 and R1 resection (11). For G1/G2 patients with Ki-67 ≤20%, relevant studies have shown a survival benefit from radical resection. However, there is still controversy regarding patients with G3 (Ki-67 >20%) liver metastases. Contrary to the ENETS, the European Society of Medical Oncology (ESMO) guidelines do not recommend G3 with LMs as a contraindication to surgery (14). But, the NANETS guidelines suggest that surgical resection may be considered for those who can achieve radical resection after adequate evaluation (15). In a Norwegian multicenter retrospective cohort study about pNET G3 combined with distant metastases (all with liver metastases), 12 patients underwent resection of the primary and metastatic lesions. 78 patients received palliative chemotherapy, which showed that patients in the surgical resection group had a better prognosis than those in the palliative chemotherapy group (3-year OS rate: 69% versus 17%, P<0.01) (16). For neuroendocrine carcinoma (NEC, G3, Ki-67 >20%), traditional views hold that surgery confers limited benefit, but recent studies have challenged this notion. Ammann et al. conducted a retrospective analysis of G3 NETLM and NEC liver metastases (NECLM), showing that some NEC patients had a median OS of 2.4 years after surgery, with tumor diameter and number of metastases identified as independent prognostic factors. This study suggests that surgery may be part of comprehensive therapy for NEC patients with localized lesions, though strict patient selection criteria are required (17). Notably, a Chinese cohort study found that elevation of Ki-67 in metastases relative to primary tumors was an independent predictor of poor OS (HR=1.396), highlighting the need to integrate Ki-67 assessment into surgical decision-making for NETLMs (18).

Furthermore, the results of previous retrospective studies and meta-analysis suggested that simultaneous resection could prolong the OS and progression-free survival (PFS) of patients (19). However, the difference in the incidence of major complications and 30-d perioperative mortality between the simultaneous resection and heterochronous groups was not statistically significant according to the results of a study (20). Although radical resection was ideal, almost all patients suffered from recurrence. Mayo et al. followed up 339 patients with NETLM who underwent radical surgical resection and found that 94% of patients had recurrence within 5 years (13). Sarmiento et al. statistically analyzed 170 NETLM patients with postoperative data and discovered a 5-year recurrence rate of up to 76% even with pathologically confirmed R0 resection (21). Radical resection is only feasible in 5%-15% of patients due to the majority of patients having bilobar metastases that cannot be completely resected (21, 22). For patients with type III and partial type II liver metastases who cannot undergo radical resection, cytoreductive surgery is an alternative treatment that can improve patients’ quality of life and prognoses. In a small sample study, resection to 70%, 90%, and 100% of the tumors did not have a significant effect on the patients’ prognoses (10). However, the comparison between radical and cytoreductive surgery was reported in a meta-analysis that included 11 studies (1,729 patients), founding that cytoreductive surgery was associated with a significantly shorter OS, with a risk ratio of 3.49 (95% CI, 2.70-4.51; p<0.001) (23).

Liver transplantation (LT) can be performed when patient is subject to Milan criteria, i.e., absence of extrahepatic lesions; histologically high-differentiated NETs (G1-G2, Ki67<10%); resectable primary tumor; metastatic load <50% of total liver volume; stable disease for at least 6 months prior to transplantation; and age <60 years (24). ENETS also published the criteria for patients to be eligible for LM, including highly differentiated low-grade diseases and no extrahepatic diseases (7). In addition, the UK has conducted a major pilot project in the field of liver transplantation, which was initiated by the Liver Advisory Team of the National Health Service (NHSBT) to comprehensively assess and recommend indications, patient selection criteria, etc., for liver transplantation. The clinical trials NCT02878473, NCT04195503, and NCT04556214 are currently ongoing. These trials focus on early-stage intrahepatic cholangiocellular carcinoma (iCCA), primary or recurrent iCCA, and locally advanced disease requiring downstaging, respectively. The results of the studies will help demonstrate the benefits of liver transplantation for disease treatment (25). Evaluation of these criteria requires careful imaging such as CT, MRI and PET-CT (26). Studies have shown that the 5-year OS and disease-free survival (DFS) rates of LT patients are 47%-71% and 20%-32%, respectively (10). However, the length of pre-transplantation observation, threshold of Ki-67 index, shortage of organs, and high recurrence after LM also limit the wide use (27).

For initially unresectable NETLM, a staged strategy of “downstaging therapy + surgery/LT” can be considered. Local therapies like TACE and PRRT can reduce tumor burden, converting some patients to resectable status or meeting LT criteria (28, 29). A Dutch study showed that oligometastatic patients (<3 lesions) treated with neoadjuvant 177Lu-octreotide had improved surgical resection rates and a median PFS of 69 months, suggesting that staged treatment may improve survival (30).

### 3.2 Local treatment

Local treatments can be equally beneficial for patients. The most common treatment modalities include ablation, TAE, TACE, and SIRT. Ablation generates thermal energy within the tumor tissue to cause coagulative necrosis, leading to cellular protein denaturation, which is indicated for smaller lesions and can be used as an adjunct to surgery. Usually 70-90% cellular attenuation can be achieved, mainly including radiofrequency ablation (RFA) and microwave ablation (MWA) (31). Akyildiz et al. retrospectively studied 89 patients with NETLM treated with ablation and found significant symptom control in 97% of patients. The median PFS (mPFS) time for patients treated with ablation alone was up to 15 months (32). A systematic review of ablation for NETLM showed 92% improvement in symptoms after RFA, with a median duration of symptom relief of 14–27 months (33). Ablation can also be used during the transition period to slow disease progression in multifocal unresectable disease requiring systemic or pharmacologic therapy. Microwave ablation’s ability to penetrate tissues less susceptible to the “heat sink” effect may improve the efficacy of treatment for larger or irregularly shaped lesions, which makes it possible to treat NETLM with complex anatomical locations (34, 35). Pickens et al. found that MWA combined with or without surgical resection was associated with clinical improvement in 95% of patients with a 5-year OS rate of 70% (36). TAE is another commonly used local therapy to effectively control tumor growth and improve symptoms in patients with NETLM. Since NETLM vascularization is mainly dependent on the arterial system, the normal liver is primarily supplied by the portal venous system (37). In a cohort of 160 patients, Zener found 1-, 3-, and 5-year OS of well to moderately differentiated patients to be 87%, 59%, and 48%, respectively. Among them, complete response (CR) was 13%, partial response (PR) was 40%, and stable disease (SD) was 24% (38). Among the 84 patients evaluated by Strosberg et al, 23 underwent imaging follow-up; 48% had PR, 52% had SD, and DCR was 77-100% (39). Besides, Guerbet (i.e., conventional TACE (cTACE), and chemotherapeutic agents mixed with iodinated poppy seed oil or drug-eluting beads (DEBs-TACE) can also be used. The aim is to increase the local concentration of cytotoxic drugs in the tumor and decrease the systemic concentration and adverse effects. However, the use of DEBs-TACE in non-cirrhotic patients was associated with an increased risk of biliary tract injury and hepatic infarction due to the lower portal blood supply and higher arterial blood supply in non-tumorigenic livers [odds ratio (OR)=6.628 (95% confidence interval (CI): 3.699-11.876), P<0.001; OR=35.2 (95%CI: 8.41-147.36), P<0.001) (40, 41). The drugs used for TACE are similar to the systemic chemotherapeutic agents used for primary NET. Of these, doxorubicin or streptozotocin are the most commonly used (42). TACE is mainly applied to NETLM patients who are unwilling to accept or unable to undergo surgery due to some reasons, such as advanced age, insufficient hepatic functional reserve, high-risk tumor sites, or bridging and down-staging treatment for patients with liver transplantation in the waiting period. Adverse effects associated with TAE and TACE simultaneously warrant attention. Post embolization syndrome is usually associated with vomiting, abdominal pain and fever. Serious cases may result in hepatic necrosis, liver abscesses, ischemic cholecystitis, and even death (43). In contrast, SIRT is the transarterial deposition of a radioactive source within a tumor, which is primarily a proximal radiation therapy that destroys tissue and blocks blood flow to a slight degree. Compared with TACE, the incidence of postembolization syndrome is lower, and SIRT is mostly a one-time treatment that can be given on an outpatient basis, thus eliminating the need for hospitalization. SIRT can be used for salvage therapy and tumor shrinkage in diffuse NETLM. Most SIRT uses yttrium 90 (90Y) without environmental radiation (44). Another device loaded with Holmium-166 (166Ho) has recently become available. Holmium is characterized by a high degree of paramagnetism, which can be quantified by MRI, and has the advantage that a small fraction of the gamma rays can be used for nuclear imaging (45). Frilling et al. recently conducted a meta-analysis of 27 retrospective studies on 90Y microsphere radioembolization for NETLM. Objective response rate (ORR) and disease control rate (DCR) were 51% (95%CI: 47-54%) and 88% (95%CI: 85-90%), respectively. 1-, 2-, and 3-year survival rates were 95%, 87%, and 78%, respectively, with a median OS of 57 months (46). Similar results were found in a study by Jia et al. that a systematic review of 11 studies showed a DCR of 86% (range: 62.5-100%). 1-, 2-, and 3-year survival rates were 72.5%, 57%, and 45%, respectively. The median OS was 28 months (range: 14–70 months) (47). Notably, the unique complication is acute radiation injury. Radiation pneumonitis characterized by diffuse interstitial changes can be induced in 1–6 months when hemophagocytic syndrome (HPS) >10% (48). Secondly, hepatic sinusoidal obstruction syndrome characterized by jaundice and ascites can emerge 4–8 weeks after SIRT. Death can occur in severe cases, with a high mortality rate of up to 30% (49, 50).

### 3.3 Somatostatin analogues

Octreotide and lanreotide, somatostatin analogues (SSAs), were administered every 10 or 14 days, which were adopted into clinical practice in the late 1980s and mid-1990s, respectively (4). SSA has anti-hormone secretion and tumor growth inhibiting effects, and is applicable to NET patients with slow growth, Ki-67 ≤ 10%, and SSTR positivity. Small sample study confirmed that the use of SSA in pNETs with Ki-67>10% could also prolong PFS of patients (51). The CLARINET study showed favorable efficacy of lanreotide in pancreatic or extra-pancreatic NET. The PROMID study established long-acting octreotide as a therapeutic position in better differentiated midgut NET patients (52). Them can be administered once a month for greater convenience (53). PFS was significantly prolonged in better differentiated midgut NET patients treated with long-acting octreotide with a favorable safety profile (54). In addition, octreotide auto-injector pens and another formulation of octreotide for subcutaneous self-release (CAM2029) are in development (55). Currently, oral octreotide is mainly used for the treatment of philtrum hypertrophy, and relevant studies include the CH-ACM-01 trial, the OPTIMAL trial, and the MPOWERED trial (56–58). Other therapeutic methods include oral nonpeptide growth inhibitor type 2 receptor agonists and drugs such as somatoprim, which has an affinity for SSTR (59, 60). These innovative therapeutic approaches may contribute to more effective treatment of patients with NET/NETLMs.

### 3.4 Systemic therapies

Conventional chemotherapy drugs can cause tumor cell death via interfering with the DNA synthesis or transcription process, including temozolomide, streptozotocin, etoposide, and platinum, which are mainly indicated for NETs with high proliferative index. Rycke et al. conducted a retrospective study of 62 patients with advanced pNET from three centers in France, and the results confirmed that the treatment modality of temozolomide or streptozotocin in combination with 5-FU or capecitabine is safe and feasible, and is well tolerated by patients, with a PFS of 9.2 months (61). The results of the phase II ECOG-ACRIN E2211 study of temozolomide combined with capecitabine (CAPTEM) versus single-agent temozolomide (TEM) for the treatment of advanced pNETs demonstrated a significant increase in PFS for the combination chemotherapy group (22.7 months vs. 14.4 months, P=0.022), and the low expression level of MGMT was significantly correlated with the efficacy of temozolomide, however there was no statistically significant difference between the ORR of the two groups (CAPTEM: 40% vs TEM: 34%) (62). STEM study showed that temozolomide combined with teguio for advanced pNETs had an ORR of 36.7% and patients with low MGMT expression had a higher ORR after receiving treatment (63).

Sunitinib is a multi-targeted tyrosine kinase inhibitor (TKI) that inhibits the activity of multiple receptors. In a study of patients with locally advanced or metastatic pNETs, sunitinib prolonged patients’ median PFS from 5.8 to 12.6 months and OS from 29.1 to 38.6 months (64). Everolimus is a targeted agent against the mTOR signaling pathway. In the RADIANT-3 study, everolimus significantly prolonged PFS of patients with pNETs from 4.6 to 11 months, with a 65% reduction in the risk of progression or death (65). In the RADIANT-4 study, the drug similarly demonstrated therapeutic efficacy in patients with non-functional pulmonary and gastrointestinal (GI) NETs, extending median PFS from 3.9 to 11 months with a 52% risk reduction (66). Sofantinib has dual antiangiogenic and immunomodulatory activities and exerts antitumor effects through inhibition of VEGFR1 to 3, FGFR1 and CSF-1R kinase activities (67, 68). The drug is approved for locally advanced or metastatic, progressive non-functional and well-differentiated (G1, G2 grade) ep-NET that cannot be surgically resected. In two studies led by Chinese investigators, the SANET-ep and SANET-p studies provided evidence-based medical evidence for the treatment of NETs. The SANET-ep study in patients with ep-NET showed that the mPFS for the primary study endpoint in the sofacitinib arm was more than twice as long as in the placebo arm (9.2 vs. 3.8 months) (69). The SANET-p study in patients with pNET showed an mPFS of 10.9 months in the sofantinib group and 3.7 months in the control group. In further imaging evaluations, the mPFS was 13.9 months in the sofacitinib group compared to 4.6 months in the control group (70). Cabozantinib is a small-molecule, multi-target oral TKI. the CABINET study evaluated cabozantinib versus placebo in 298 patients with NET whose disease progressed after treatment. the mPFS in the cabozantinib arm was 8.4 months versus 3.9 months in the placebo arm for the 203 ep-NET (P<0.001). In the other 95 patients with pNET, the mPFS was 13.8 months in the cabozantinib group and 4.4 months in the placebo group (P<0.001). ORR was 5% and 19% between ep-NET and pNET, respectively (71). Another phase II TALENT study included lenfatinib in 111 cases of advanced G1-G2 pNET and GI-NET that had progressed after targeted or SSA therapy. The results showed that ORR between the two groups was 44% and 29.9%, the median duration of regression was 19.9 and 33.9 months, mPFS was 15.6 and 15.7 months, respectively (72). In addition, although the immune drugs are highly acclaimed, they do not perform well in tumors. The study by Vijayvergia et al. suggests that pembrolizumab may be safe for use in patients with G3 NETs, especially those with dMMR/MSI-H, but monotherapy activity is limited.

### 3.5 PRRT

PRRT is a nuclide-targeted therapy based on the enriched expression of SSTR by NETs, which uses a nuclide-labeled SSTR agonist or antagonist to allow the rays to act directly on tumor cells to kill them. Currently, nuclides commonly include 177Lu, 90Y, and 111In. In 2018, the U.S. Food and Drug Administration (FDA) also formally approved PRRT for treatment of progressive growth inhibitor receptor-positive GEP-NETs. The NETTER-1 clinical trial demonstrated significantly longer PFS of patients treated with PRRT in combination with SSA compared to patients treated with SSA incremental therapy. Median survival was prolonged by 11.7 months, with a 20-month PFS of 65.2% vs 10.8% (73). However, this study was a second-line treatment and also did not include well-differentiated high-grade NET-G3 patients. Recent results on the NETTER-2 study demonstrated the efficacy of 177Lu as first-line treatment in patients with advanced G2/G3 GEP-NETs. 177Lu in combination with standard-dose octreotide LAR significantly improved patients’ ORR (43% vs. 9.3%), reduced the risk of disease progression or death compared to the increasing-dose octreotide LAR group (72%), and prolonged the PFS time in patients with first-diagnosed advanced G2/G3 GEP-NETs (22.8 months vs 8.5 months) (74). Doyle et al. included 36 patients initially treated with 90Y resin microspheres from 2013 to 2022. According to RECIST 1.1, the results showed that 36 patients had an ORR of 75% (CR 19%, PR 56%), a DCR of 97%, and a median follow-up of 581 days. According to mRECIST, 32 patients had an ORR of 85% (CR 39%, PR 45%), a DCR of 97%, and a median follow-up time of 491 days. PRRT can be used in locally advanced NF-pNET, even in tumors with limited distant metastases (oligometastases), which may also be a promising therapeutic approach. Satapathy et al. by including 40 patients with advanced inoperable/metastatic NETs treated with PRRT, suggested that 12/40 (30%) achieved PR and 22/40 (55%) were SD (75). For non-surgical, highly to moderately differentiated metastatic NETs, the study by Hamiditabar et al. included 143 patients who underwent PRRT (177LuDOTATATE), and the results suggested an ORR of 9.09%, DCR of 59.09%. The prolongation of the treatment cycle was able to provide benefit to patients (ORR: 28.57%, DCR: 85.71%) (76). In 2018, a clinical study by Partelle et al. retrospectively included 23 patients who underwent PRRT followed by surgical resection versus those who underwent direct surgical resection. The results showed a higher R0 resection rate and lower lymph node positivity in the PRRT group. In addition, the COMPOSE trial (NCT 04919226) comparing PRRT combined with chemotherapy versus everolimus in G2 to G3 GEP-NET is ongoing.

## 4 Discussion

Finally, NETLMs are a class of diseases with high complexity and heterogeneity. Regarding the drug selection, many factors need to be considered, including the primary site of the tumor, pathological grade, hormone secretion, stage, load, growth inhibitory receptor expression, blood supply, and residual function of the liver, and so on. It involves the cooperation of several departments such as surgery, medical oncology, imaging, intervention and pathology. Therefore, the model of multidisciplinary treatment has been proven to be effective. Malcolm et al. reported in 2020 that 30 pNET patients were preoperatively treated with capecitabine combined with temozolomide (CAM-TAP), of which 26 patients underwent surgery with a median PFS of 28.2 months and a 5-year survival rate of 63% (77). Dutch study noted that after 29 patients with oligometastatic (<3) pNET in the liver received neoadjuvant PRRT with 177Lu-octreotide, 9 patients underwent surgical resection, with a mPFS of 69 months, compared to 25 months for the other 90 patients who had >3 hepatic metastases as controls (30). In 2022, Liu et al. included 116 patients with G1/G2 NETLM treated with TAE in combination with octreotide LAR. The results showed that the mPFS under the combination regimen was 13.6 months. Liver metastatic load >50%, Ki-67 >10% and bone metastasis were independent prognostic factors for PFS; while TAE was more efficacious in patients with Ki-67 ≤10%, no bone metastasis, and well-defined liver metastatic tumor (78). In the CLARINET study, PFS was halved in patients with liver metastatic load >25% compared to those with ≤25% (24.1 vs 50.8 months) (79). The GETNE-TRASGU study also found that a hepatic metastatic load >50% was a factor negatively associated with PFS for SSA therapy, whereas a hepatic metastatic load ≤25% was a factor positively associated with PFS for SSA therapy (80). A study evaluated the efficacy of SIRT combined with PRRT versus SIRT alone in patients with liver-dominant NETs. The results showed no statistically significant difference in OS or PFS between the two groups, but the combination treatment improved survival (67.5 months vs 34.9 months) (81). Another study evaluated CAPTEM in combination with LuTate PRRT versus PRRT alone in patients with mNETs. Results showed that the 15-month PFS rate in the CAPTEM/PRRT group was similar to the PRRT-alone group, but the combination group was more toxic. (ACTRN12615000909527) Li et al. compared the clinical efficacy of sufatinib combined with TAE versus sufatinib alone in the treatment of NEMLTs, which is still ongoing (82).

Therefore, it is still controversial whether to adopt the traditional systemic and then local treatment, or local and then systemic treatment and systemic combined with local treatment for patients with NETs with high tumor load. In addition, there is a lack of high-level evidence-based medical evidence for NETLMs. The choice of treatment, indications for combination therapy, and evaluation of the efficacy for liver metastases are all urgent issues. Notably, systemic inflammatory response markers such as neutrophil-to-lymphocyte ratio (NLR), platelet-to-lymphocyte ratio (PLR), and cytokines (e.g., IL-8, VEGF) show potential in prognostic assessment of NETLM. Giannetta et al. indicated that elevated NLR and PLR are associated with poor prognosis in NET patients, serving as simple indicators for predicting disease progression and survival. Additionally, the immunomodulatory role of PD-1/PD-L1 expression in the tumor microenvironment provides a theoretical basis for immunotherapy, though more studies are needed to validate its clinical value (83). In contrast to the systematic review by Muttillo et al., this study places greater emphasis on individualized treatment strategies for NF-NETLM. Future research should focus on sequential strategies integrating local and systemic therapies within multidisciplinary team (MDT) frameworks, precision stratification based on molecular markers (such as Ki-67 and PD-L1), and combinatorial applications of novel targeted agents (like dual-target inhibitors) with immunotherapies. Additionally, the role of liver transplantation in unresectable NETLM requires validation through prospective studies, particularly regarding optimization of the Milan criteria and strategies for preventing postoperative recurrence (84). With the development of genomics, spatial transcriptomics, and proteomics, more therapeutic modalities and broader drug development are important trends for the future. We look forward to investing more research for neuroendocrine tumors and liver metastases in the future.

## 5 Conclusion

This review retrospectively examines and analyzes a substantial literature and details the therapeutic approaches to NETLMs. Surgery is the primary option for the treatment of NETLMs but suffers from the problem of postoperative recurrence. Non-surgical treatments are diverse, including TAE, TACE, SIRT, SSAs, pharmacologic therapies, and PRRT, which each has its own indications and efficacy. Multidisciplinary treatment has been applied to improve patients’ outcomes and survival. However, there is still a lack of high-level evidence-based medical evidence for the treatment of NETLMs. Issues regarding the choice of treatment regimen, indications for combination therapy, and disease prognosis urgently warrant resolving. Further exploration and optimization of therapies are needed in the future to improve the survival quality and prognosis of patients.
