# Supplementary material for: Management of liver metastases from non-functional gastroenteropancreatic neuroendocrine tumors: a systematic review
Source: Front Endocrinol (Lausanne). 2025 Jul 24;16:1601185. doi: 10.3389/fendo.2025.1601185 (PMC12328144; doi:10.3389/fendo.2025.1601185)
Supplement: Supplementary file 1 [file Table1.docx]

**The detail of search strategy**

**PubMed** 326 ((Neuroendocrine Tumor[Title/Abstract]) OR (Neuroendocrine Neoplasm[Title/Abstract])) AND (Liver Metastasis[Title/Abstract])

**Embase** 471 ('neuroendocrine tumor':ab,ti OR 'neuroendocrine neoplasm':ab,ti) AND 'liver metastasis':ab,ti

**Cochrane** 1100

#1 (Neuroendocrine Tumor):ti,ab,kw 1441

#2 (Neuroendocrine Neoplasm):ti,ab,kw 234

#3 (Liver Metastasis):ti,ab,kw 4475

#4 #1 OR #2 1465

#5 #4 AND # 3 1100
